# Surgery for Complex vs. Simple Native Left-Sided Endocarditis: Insights from an Extended Follow-Up on Survival, Recurrent Infection, and Valve Durability

**DOI:** 10.3390/jcm14165870

**Published:** 2025-08-20

**Authors:** Reut Shavit, Katia Orvin, Hila Shaked, Victor Rubchevsky, Yaron Shapira, Ran Kornowski, Ram Sharony

**Affiliations:** 1 Department of Cardiothoracic Surgery, Carmel Medical Center, Haifa 3436212, Israel; 2 Department of Cardiology, Rabin Medical Center, Petah Tikva 4941492, Israel; 3 Gray Faculty of Medical and Health Sciences, Tel Aviv University, Tel Aviv 6997801, Israel; ramsha59@gmail.com; 4 Infectious Disease Unit, Rabin Medical Center, Petah Tikva 4941492, Israel; 5 Department of Cardiothoracic Surgery, Rabin Medical Center, Petah Tikva 4941492, Israel

**Keywords:** infective endocarditis, root abscess, perivalvular extension, long-term survival, reoperation, mechanical valves, valve durability, mitral valve repair

## Abstract

**Background/Objectives**: We compared short- and long-term outcomes of patients with native left-sided infective endocarditis (IE) confined to the valve leaflet (“simple”) versus those with perivalvular extension (“complex”) over two decades. **Methods**: From 2005 to 2024, 177 patients (mean age 59.6 ± 13.8 years, 71.8% male) underwent surgery for IE. Patients were classified as having simple (*n* = 129) or complex IE (*n* = 48) based on imaging and intraoperative findings. Mean follow-up was 86.5 ± 63.3 months (range: 2–232 months). Outcomes included operative and late mortality, recurrent infection, and reoperation. **Results**: Complex IE was associated with worse preoperative status, longer ICU stays, and mechanical ventilation times. Predictors of early mortality included critical preoperative state (OR 6.35, *p* = 0.001), chronic renal failure/dialysis (OR 3.01, *p* = 0.05), and staphylococcal IE (OR 5.62, *p* = 0.002) but not perivalvular extension. Overall survival at 1, 5, 10, 15, and 20 years was 83%, 74.2%, 59.9%, 51.3%, and 40.7%, with no significant difference between groups (*p* = 0.18). Female gender (HR 1.93, *p* = 0.04) and chronic renal failure (HR 3.5, *p* < 0.01) predicted late mortality. Freedom from re-endocarditis and reoperation d/t relapse of endocarditis was 94.2% and 97.3%, respectively. Freedom from re-intervention d/t structural valve degeneration was 92.1% at 10 years. Repair was performed in 28.2% of cases involving the mitral valve, with 93.1% freedom from reoperation. **Conclusions**: Surgery for complex IE is not an independent risk factor for long-term mortality. Rates of recurrent endocarditis and reoperation are remarkably low. Excellent durability of bioprostheses and mitral repair was demonstrated.

## 1. Introduction

Infective endocarditis (IE) presents a significant public health challenge. In 2019, the incidence of IE was estimated at 13.8 cases per 100,000 people annually, and it was responsible for 66,300 deaths worldwide [1]. Surgery is undertaken in 40% to 50% of patients with IE, with three principal indications: valve dysfunction leading to heart failure, uncontrolled infection, and prevention of embolism [2]. We have previously reported on our single-center surgical outcomes for native left-sided endocarditis [3]. We categorized patients based on the extent of infection, defining “simple” endocarditis as cases limited to the valve leaflets without involvement of surrounding structures and “complex” endocarditis as cases with perivalvular extension, such as abscesses, fistulas, or pseudoaneurysms. The objective of the current study was to compare long-term surgical outcomes between patients with simple and complex native left-sided endocarditis. We hypothesized that patients with complex endocarditis, characterized by perivalvular extension, would have worse outcomes—including higher mortality and re-intervention rates—compared to those with simple valve-confined disease. This updated comparative dataset, which includes additional patients and extended follow-up of up to 20 years, provides further insights into surgical outcomes, including survival rates, incidence of re-endocarditis, valve durability and the need for re-interventions, as well as guidance on the choice of prosthesis, valve repair in endocarditis, and independent risk factors for early and late mortality.

## 2. Materials and Methods

### 2.1. Study Population

Between 2005 and 2024, a total of 334 patients underwent surgery for IE at our center. Exclusion criteria were prosthetic valve endocarditis (*n* = 100), right-sided endocarditis (*n* = 11), reoperation (*n* = 16), or uncertain diagnosis (*n* = 30). The overall patient cohort in this study included 177 consecutive patients with native left-sided IE who underwent surgery.

### 2.2. Data Collection

Methods for data collection, surgical classification, and follow-up were similar to those outlined in our earlier study [3]. In short, patient characteristics, operative and postoperative data, and the development of complications during follow-up were retrospectively collected. Early and late echocardiographic data were also recorded. These data were used to analyze the association between various patient and disease characteristics and the perioperative, intermediate, and long-term outcomes.

### 2.3. Study Endpoints

The primary endpoints were operative and long-term mortality. Secondary endpoints were recurrent IE, the need for late valve surgery for recurrent endocarditis, re-intervention for structural degeneration, and recurrence of mitral regurgitation post-repair.

### 2.4. Definitions

Complex disease was defined as perivalvular destruction, such as abscess or fistula, that required reconstruction. Abscess was defined as an abnormal echolucent or echodense inhomogeneous area in the peri-annular region or mitral-aortic continuity. Pseudoaneurysm appeared as a pulsatile, echo-free space with blood flow on color Doppler. Fistula was diagnosed by color Doppler, with evidence of abnormal communication between two cardiac chambers. Leaflet perforation was identified as a discontinuity in endocardial tissue traversed by Doppler flow. Vegetations were filamentous or pedunculated masses attached to valve structures. In the mitral position, complex endocarditis included involvement of the posterior annulus or aorto-mitral continuity. Isolated leaflet perforation or vegetation confined to the leaflet was classified as simple endocarditis. Critically ill patients were patients presenting with cardiogenic shock or multisystem failure or requiring mechanical ventilation. Operative mortality was defined as mortality in the index hospitalization or within 30 days. IE was diagnosed based on the latest guidelines [2]. All other morbidity parameters were defined according to the Society of Thoracic Surgeons database criteria [4]. The severity of valve disease was assessed using echocardiography, color Doppler flow, and spectral Doppler as per guidelines [5] and was graded on a scale of 0 to 3 (none/trivial, mild, moderate, severe). The postoperative intravenous antibiotic regimen was determined according to bacterial sensitivities and was given for a period of 4–6 weeks post-op as per infectious disease consult based on the last positive bacterial culture and intra-operative culture results.

### 2.5. Surgical Management

**Figure 1:**
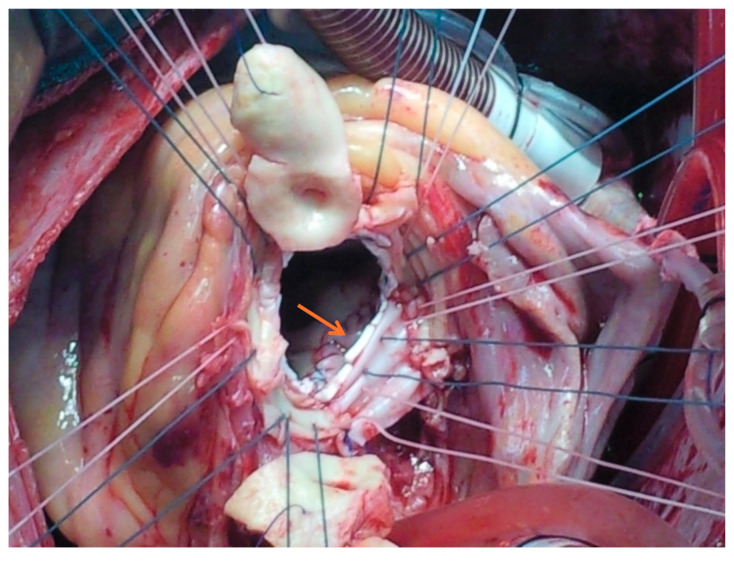
Repair of an aortic root abscess using a xenopericardial patch during aortic root replacement for complex endocarditis with perivalvular involvement. Arrow: Aorto-mitral curtain patch.

The general operative principle was radical debridement of all the necrotic and grossly infected tissue with subsequent assessment and reconstruction using single or multiple xenopericardial patches, as deemed necessary (Figure 1).

Whenever feasible, mitral valve repair was preferred over replacement, and techniques used were triangular resection, reconstruction of the valve leaflets and perivalvular area when indicated using xenopericardial patch, and ring annuloplasty.

### 2.6. Statistical Analysis

The results are reported as means and SDs or median and interquartile range, as appropriate. Categorical variables were compared using the chi-square test or Fisher’s exact test and Mann–Whitney U test, and continuous variables using Student’s *t* test. The associations between the clinical characteristics and endpoints were studied separately using survival analysis. The cumulative percentages for survival during different time periods were estimated using the Kaplan–Meier technique. Differences in survival were tested using the log-rank test. Factors associated with early and long-term mortality were analyzed using multiple logistic regression and Cox proportional hazard models. Variables were included in the multivariate models if they had a *p* value < 0.05 in univariate analysis and/or were deemed clinically relevant based on prior literature and expert consensus. Differences between groups were quantified by hazard ratios and 95% confidence intervals. Two-sided *p* < 0.05 was considered statistically significant. Statistical computations were carried out using IBM SPSS Statistics software version 29.0.2.0.

## 3. Results

### 3.1. Patient Cohort and Disease Classification

Between January 2005 and July 2024, 177 patients underwent surgery for primary native left-sided valve infective endocarditis. Of these, 48 (27%) were in the complex group while 129 (73%) were in the simple group. The clinical characteristics of the patients are detailed in Table 1.

Patients with complex disease exhibited significantly higher rates of diabetes mellitus, acute renal failure, dialysis dependency, atrioventricular block, elevated white blood cell counts, increased creatinine levels, and lower hemoglobin values. Aortic valve involvement was significantly more common in the complex group compared to the mitral valve, with higher rates of double-valve involvement also observed. The most common primary indication for surgery was CHF/valve dysfunction in 66.7% of patients.

Complete follow-up was available for all patients, with a mean duration of 86.5 ± 63.3 months (range: 2–232 months). Immediate and late postoperative outcomes are summarized in Table 2.

### 3.2. Early and Long-Term Survival

The overall operative mortality rate was 11.9% (*n* = 21). Higher operative mortality was observed in the complex group (*n* = 9, 18.8%) compared to the simple group (*n* = 12, 9.3%, *p* = 0.08). Patients in the complex group had significantly longer intensive care unit (ICU) stays and required prolonged mechanical ventilation more frequently. Univariate analysis identified several factors significantly associated with higher early mortality, including preoperative critical state (52.4% vs. 14.1%, *p* < 0.01), staphylococcal IE (47.6% vs. 13.5%, *p* < 0.01), chronic renal failure (38.1% vs. 14.1%, *p* = 0.01), age > 65 years (61.9% vs. 39.7%, *p* = 0.05), and diabetes mellitus (42.9% vs. 21.8%, *p* = 0.03). In addition, patients whose indication for surgery was uncontrolled infection had higher operative mortality compared to patients with other indications, mainly CHF, which was the most prevalent primary indication for surgery (*p* = 0.002). Other variables, including gender, recent stroke, left ventricular function, valve position, double-valve involvement, and adjunct procedures, were not significantly associated with operative mortality. A multiple logistic regression analysis identified preoperative critical state (OR = 6.75, 95% CI: 2.35–19.34, *p* < 0.001), chronic renal failure/dialysis (OR = 3.01, 95% CI: 1.001–9.07, *p* = 0.05), and staphylococcal IE (OR = 5.45, 95% CI: 1.87–15.89, *p* = 0.002) as significant independent predictors of early mortality.

**Figure 2:**
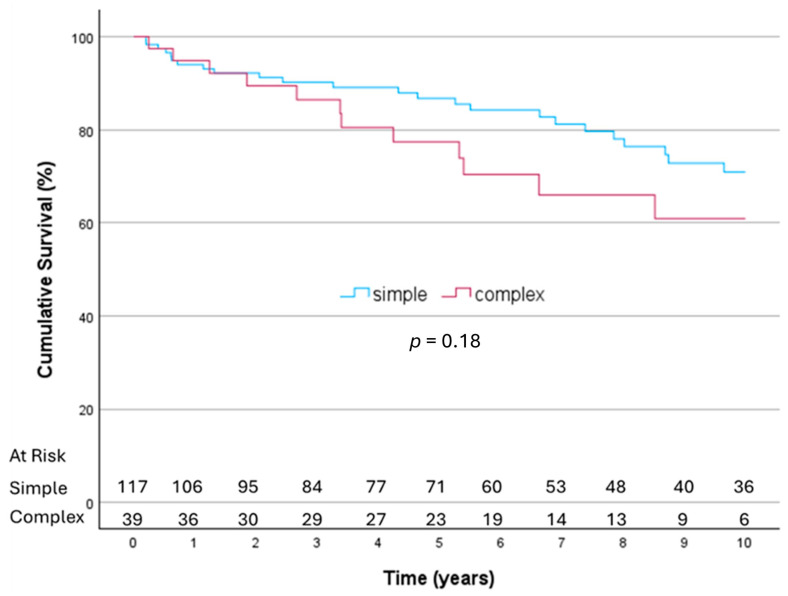
Long-term survival analysis for complex versus simple endocarditis at 10 years (operative mortality excluded).

Among the 44 patients who died during follow-up, cardiac-related causes accounted for 18.1% of deaths, while non-cardiac causes accounted for 61.4%. The cause of death was uncertain in 20.5% of deaths. Overall survival rates were 83%, 74.2%, 59.9%, 51.3%, and 40.7% at 1, 5, 10, 15, and 20 years, respectively. Differences in long-term survival, however, were not significant between patients with simple and complex disease (Figure 2).

Univariate analysis revealed better long-term survival among patients receiving mechanical valves compared to those with tissue valves (96.4% vs. 92.1%, 96.4% vs. 78.2%, 89% vs. 54%, 89% vs. 40%, and 59.3% vs. 24% at 1, 5, 10, 15, and 20 years, respectively, *p* < 0.01). Multivariate analysis for long-term survival demonstrated that age > 65 years (*p* = 0.015, HR 2.38), female gender (*p* = 0.042, HR 1.93), and chronic renal failure (*p* < 0.01, HR 3.56) were independent risk factors for long-term mortality (Table 3). Neither the use of mechanical valves nor the extent of infection (“complex disease”) was independently associated with long-term mortality.

### 3.3. Re-Endocarditis

During the follow-up period, four cases of re-endocarditis were reported: two in the complex group and two in the simple group (*p* = 0.17). However, only one was due to relapse with the same pathogen. Cumulative freedom from re-endocarditis was 94.2%, and from relapse endocarditis, it was 98.7%. One patient with complex IE required reoperation for recurrent endocarditis 12 years postoperatively due to a different pathogen, resulting in an overall freedom from reoperation for endocarditis of 97.3%.

### 3.4. Structural Valve Degeneration

**Figure 3:**
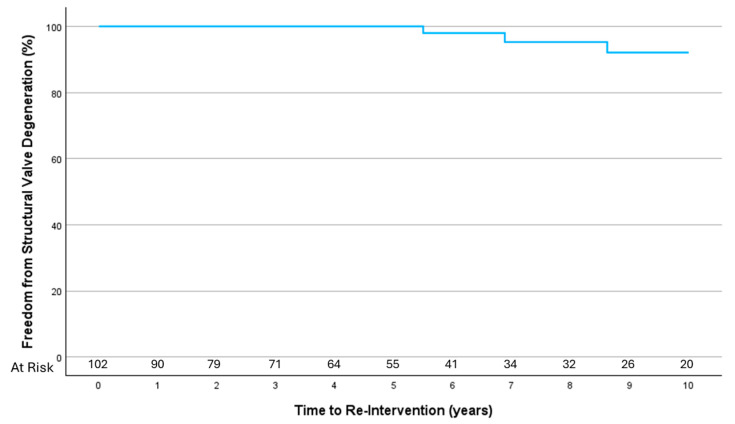
Freedom from structural valve degeneration at 10 years.

Echocardiographic follow-up data were available for 116 patients (74% of survivors), with a mean follow-up duration of 75.6 ± 63.7 months. Overall, 14 patients (8.9%) underwent repeat cardiac procedures (see Appendix A Table A1). Of these, six patients underwent re-intervention for structural valve degeneration after a mean of 9.8 ± 3.5 years (one re-do surgical mitral valve replacement, four mitral valve in valve, and one aortic valve in valve implantation). Overall freedom from re-intervention d/t structural valve degeneration was 84.5% and 95.5% at 10 years (Figure 3).

### 3.5. Mitral Valve Repair

Mitral valve repair was performed in 31 cases (28.2%), while replacement was performed in 79 cases (71.8%) of all cases involving the mitral valve. These included 25 cases of active IE and 6 cases of healed IE. Six patients with complex disease underwent mitral valve repair (19.4%). Late echocardiographic follow-up was available for 24 patients (77.4%) who underwent mitral valve repair, with a mean follow-up duration of 102 ± 81.2 months. Severe mitral regurgitation occurred in three patients, all from the simple group: Two patients experienced early postoperative recurrence of mitral regurgitation after initially favorable intraoperative echocardiographic results and subsequently required mitral valve replacement. Only one patient developed moderate-to-severe mitral regurgitation during long-term follow-up. Freedom from re-intervention was 93.3% post mitral repair.

## 4. Discussion

This study summarizes the short- and long-term outcomes of patients who underwent surgery for primary native left-sided IE over a 20-year period. Surgery for infective endocarditis with complex perivalvular involvement is associated with increased short-term morbidity. Within the limits of our single-center long-term dataset, there was no statistically significant difference in survival between groups. The overall rate of recurrent endocarditis and reoperation was very low during the follow-up period. Our results highlight areas of ongoing debate, particularly regarding prognostic factors that can guide decision-making for critically ill patients, the choice of prosthesis, and the decision to perform valve repair versus replacement during the initial surgery.

Complex disease was associated with markedly worse baseline characteristics. Patients from the complex group experienced significantly higher rates of postoperative complications compared to those within the simple group. Mortality rates remain high despite advancements in surgical techniques and perioperative care. This finding, which aligns with most epidemiological studies on IE [1,6,7,8], is particularly notable given the relatively young patient population, with a mean age of 59.6 years. Importantly, complex disease itself was not an independent risk factor for mortality; rather, a critical preoperative state emerged as the primary determinant of prognosis. These findings suggest that identifying and aggressively managing high-risk patients, particularly those in a critical preoperative state or with staphylococcal IE, could improve early postoperative outcomes [9]. Age, female gender, and chronic renal failure, but not a “complex” disease, were identified as independent risk factors for late mortality among hospital survivors.

Most of the current understanding of outcomes in patients undergoing surgery for IE is derived from retrospective cohort studies. Said et al. [6], from the Mayo Clinic, in one of the largest single-center cohorts, reported the surgical outcomes of 801 patients who underwent surgery for IE with a mean follow-up of 4.6 years. This cohort, which included both prosthetic and native valve endocarditis, demonstrated an operative mortality rate of 8%. The overall survival rates were 68%, 45%, and 8.4% at 5, 10, and 20 years, respectively, which is slightly lower than our data. Notably, root abscesses were present in 14.2% of their patients and were identified as predictors of early mortality. In a separate study on the surgical outcomes of aortic valve endocarditis with aortic root abscess involving 143 patients, 34% were found to have an aortic root abscess [10]. The presence of an aortic root abscess was not significantly associated with higher early or late mortality but was linked to a non-significantly higher reoperation rate. Yang et al. [11] reported on 336 patients undergoing surgery for aortic valve IE, 50% of whom had root abscesses. They observed an operative mortality rate that was marginally higher in the abscess group (8.4% vs. 3.8%), although this difference was not statistically significant. However, patients in the root abscess group experienced prolonged ventilation times and longer intensive care unit stays. Ten-year survival rates were comparable between groups, with 10-year reoperation rates of 5.9% versus 7.9%. This strengthens our observation that patients in the complex group have worse presentation and operative mortality, though in the long term, survival is not impacted by the characteristics of the initial IE presentation. Also, consistent with these and additional reports, we have observed significantly more patients with aortic valve involvement in the complex IE group compared to those with mitral valve involvement.

In our cohort, LVEF was preserved in the majority of patients (normal in 87%, severely reduced in only 2.8%). This differs from our categorization of CHF as an indication for surgery, which was based on clinical decompensation due to acute valvular dysfunction, regardless of LVEF. Pizzino et al. [12] reported that IE-associated heart failure was independently associated with major adverse events—including all-cause mortality, hospitalizations, and IE relapses—over a three-year follow-up in a cohort of 102 patients with native and prosthetic valve IE. In our analysis, however, IE-related CHF was not significantly associated with long-term mortality (see Supplementary Data).

Importantly, we found that the rates of re-endocarditis and the need for re-intervention due to recurrent infection were very low, even in cases of destructive and invasive endocarditis, and except for one case, the late re-infection was caused by a different pathogen, representing re-infection but not relapse. Only one patient required reoperation for recurrent IE. This low rate of re-endocarditis can be partially explained by the low rate of IVDU in our population (2.3%), which is known to be a major risk factor for re-infection [7,13].

Although univariate analysis demonstrated that patients who underwent mechanical valve replacement have better long-term survival, this did not emerge as an independent predictor of mortality. This observation is consistent across multiple other comparative studies of mechanical versus bioprostheses [6,14]. Malmberg et al. [15] retrospectively studied the long-term outcomes of mechanical versus biological valve prosthesis in native mitral valve endocarditis and found a significant survival benefit for patients who received a mechanical valve over 12 years, with a hazard ratio of 0.4. Salazano A. et al. [16] retrospectively studied 549 patients operated on for aortic valve endocarditis between the ages of 40 and 65. A significant trend in the reduction of mechanical versus biological prosthesis implantation was observed during the study period (2000–2021). Long-term survival was significantly higher among patients receiving a mechanical prosthesis, and mechanical prostheses were associated with significantly less recurrent endocarditis after aortic valve replacement than biological prostheses.

Current guidelines [2] suggest several factors favoring a non-mechanical valve substitute in acute IE surgery, such as early surgery after a recent ischemic stroke, evidence of intracranial bleeding, women of childbearing age, high likelihood of prolonged mechanical circulatory support, advanced age and frailty, poor or unknown medical compliance, complicated postoperative courses, and patient preference. The recent trend toward bioprostheses is partly driven by valve-in-valve technologies, which have been successfully performed in some patients in our cohort, mostly for structural valve degeneration. The rate of re-intervention was remarkably low.

Structural valve degeneration is believed to be influenced by patient-related factors such as comorbidities (dyslipidemia, diabetes mellitus, metabolic syndrome, hypertension), dysregulation of phospho-calcic metabolism, and immune rejection, all of which contribute to increased leaflet mechanical stress. Additionally, prosthesis-related factors, including anti-mineralization treatment, design flaws, small prosthesis size, and severe patient–prosthesis mismatch, can lead to abnormal flow patterns [17,18]. Both patient-related factors (such as immune response) and prosthesis-related factors (such as complex implantation with abnormal flow patterns) may play a role in patients with endocarditis.

Despite these factors, our observations indicate that durability remains reasonable, with a mean time to reintervention of nearly 10 years, with most interventions occurring in the mitral position. However, the durability of bioprostheses in patients with endocarditis requires further investigation, as no comparative studies are available in the current literature.

We report that female gender was found to be an independent risk factor for late mortality. Data from the Germany-wide CAMPAIGN registry recently published [19] suggest higher risk profiles and increased early postoperative morbidity, as well as lower estimated mid-term survival among female patients undergoing IE surgery. In an analysis of 4917 patients (27% female), women presented with more comorbidities and higher surgical risk. Their early postoperative course was marked by longer ventilation times, longer ICU stays, and more frequent new-onset dialysis. While the 30-day mortality rate was similar between sexes, estimated mid-term survival was significantly higher among male patients, with female sex identified as an independent predictor of mid-term mortality.

The superiority of valve repair is well-documented in cases of degenerative mitral valve disease [20]. However, mitral valve repair in the setting of active endocarditis is particularly challenging. The principles of extensive debridement of grossly infected tissue must be meticulously upheld, and careful assessment of the remaining tissue is essential to determine the likelihood of a durable repair. Moreover, technical considerations significantly impact repair durability. Many surgeons remain reluctant to perform prosthetic annuloplasty because of the presumed high risk of re-infection, which could, in turn, significantly impair repair durability. Miura et al. [21] investigated the durability of mitral valve repair for IE associated with the location of the main infected lesion and found satisfactory durability in posterior leaflet infection without annulus invasion and in clear zone infection of the anterior leaflet. However, rough zone infections of the anterior leaflet, especially those involving multiple segments, were associated with a high risk of recurrent MR.

Mitral valve repair was successfully performed in 20% of patients with mitral valve involvement in our study, including those with active endocarditis, demonstrating encouraging durability. However, two patients experienced early in-hospital recurrence of mitral regurgitation after initially favorable echocardiographic results, emphasizing the need for careful patient selection for repair.

Several studies have reported favorable outcomes with mitral valve repair in endocarditis [22]. Helmers et al. [23] conducted a propensity-matched analysis of 267 patients who underwent mitral valve repair or replacement for isolated mitral valve endocarditis. They found better immediate outcomes, such as shorter ventilator times and reduced ICU and hospital stays, with similar 30-day and 10-year survival. Interestingly, the repair cohort was less likely to require repeat mitral valve intervention for re-endocarditis than the replacement cohort. A recently published meta-analysis of 23 studies involving 11,802 patients [24] found lower early and long-term mortality and a reduced risk of IE recurrence in patients undergoing mitral valve repair, although reoperation rates did not differ. Tomsic et al. [25] recently published data from a prospective nationwide database in the Netherlands, including adult patients who underwent primary mitral valve intervention for active endocarditis between 2013 and 2020. Of the 715 patients who met the inclusion criteria, 41% underwent valve repair. The early mortality rate was 13%. Both early mortality (7.5% vs. 16.9% for repair vs. replacement) and five-year survival (HR 2.216) were worse for patients undergoing valve replacement. Moreover, there was no significant difference in five-year freedom from mitral valve reintervention (89.9% vs. 94.1% for repair vs. replacement).

## 5. Conclusions

In conclusion, while patients with complex disease had worse preoperative conditions and higher early complication rates, long-term survival was comparable between groups. However, this should be interpreted with caution due to the small sample size. Despite the invasive nature of complex disease, the overall rate of re-endocarditis was remarkably low, and reoperation for recurrent infection was rare. Mitral valve repair, though technically challenging in active endocarditis, was successfully performed in select cases with encouraging durability. Prosthesis selection remains a complex decision that warrants further investigation, balancing the potential survival benefits of mechanical prostheses with the durability of bioprostheses and the option for future valve-in-valve procedures. These findings highlight the importance of personalized surgical strategies and early intervention for high-risk patients to optimize outcomes. Multicenter studies with larger sample sizes are required to provide more statistically robust findings.

Study limitations: This study has several limitations. First, it is a single-center study with a retrospective design. Surgeon variability in techniques, particularly in valve repair, could also introduce inconsistencies in outcomes. Comparisons for long-term survival were limited to 10 years to ensure a sufficient sample size in both groups. The lack of significance in long-term survival rates between the groups should be interpreted with caution due to the small sample size. Due to the low incidence of this particular complication (i.e., infective endocarditis complicated by perivalvular involvement), achieving a sufficient sample size to ensure 80% power is challenging. Therefore, observed trends and clinical context should also play an important role in interpreting the results, and adverse trends cannot be excluded due to a lack of power.

## Figures and Tables

**Table 1:** Patient characteristics.

Parameter ^†^		Overall (*n* = 177)	Simple (*n* = 129)	Complex (*n* = 48)	*p*-Value
Demographics					
Age, mean ± SD (years)		59.6 ± 13.8	58.56 ± 14.4	62.42 ± 11.4	0.09
Male gender, *n* (%)		127 (71.8%)	91 (70.5%)	36 (75.0%)	0.56
Comorbidities					
DM		43 (24.3%)	26 (20.2%)	17 (35.4%)	0.03
HTN		76 (42.9%)	53 (41.1%)	23 (47.9%)	0.41
COPD		10 (5.6%)	6 (4.7%)	4 (8.3%)	0.46
PVD		7 (4.0%)	6 (4.7%)	1 (2.1%)	0.67
Atrial fibrillation		30 (16.9%)	21 (16.3%)	9 (18.8%)	0.66
IVDU		4 (2.3%)	2 (1.6%)	2 (4.2%)	0.30
Previous stroke (old)		7 (4.0%)	5 (3.9%)	2 (4.2%)	0.45
Predisposing cardiac condition	Myxomatous valve	24 (13.6%)	20 (15.5%)	4 (8.3%)	0.21
	BAV	19 (10.7%)	9 (7.0%)	10 (20.8%)	0.008
	RHD	4 (2.3%)	4 (3.1%)	0 (0.0%)	0.57
	Valve stenosis/regurgitation	19 (10.7%)	15 (11.6%)	4 (8.3%)	0.53
	Other	5 (2.8%)	3 (2.3%)	2 (4.2%)	0.61
Renal Failure	Chronic	19 (11.2%)	12 (9.5%)	7 (16.3%)	0.26
	Dialysis	11 (6.2%)	5 (3.9%)	6 (12.5%)	0.07
LV dysfunction	Preserved (EF > 50%)	154 (87.0%)	115 (89.1%)	39 (81.3%)	0.20
	Mild (EF 40–50%)	16 (9.0%)	10 (7.8%)	6 (12.5%)	
	Moderate (EF 30–40%)	2 (1.1%)	2 (1.6%)	0 (0.0%)	
	Severe (EF < 30%)	5 (2.8%)	2 (1.6%)	3 (6.3%)	
Clinical presentation					
Critical state		33 (18.6%)	21 (16.3%)	12 (25.0%)	0.18
Recent Stroke		30 (16.9%)	22 (17.1%)	8 (16.7%)	0.85
Atrioventricular block		6 (3.4%)	0 (0.0%)	6 (12.5%)	<0.001
Acute renal failure		19 (10.7%)	10 (7.8%)	9 (18.8%)	0.03
Valve involved	Aortic	71 (40.1%)	46 (35.7%)	25 (52.1%)	0.014
	Mitral	90 (50.8%)	74 (57.4%)	16 (33.3%)	
	Both	16 (9.0%)	9 (7.0%)	7 (14.6%)	
WBC		10.29 ± 5.28	9.57 ± 4.09	12.22 ± 7.31	0.02
Creatinine		1.43 ± 1.61	1.19 ± 1.11	2.05 ± 2.42	0.02
Hemoglobin		10.86 ± 1.90	11.06 ± 1.94	10.34 ± 1.71	0.03
Causative agent	*S. aureus*	31 (17.5%)	20 (15.5%)	11 (22.9%)	0.25
	Strep. species	65 (36.7%)	49 (38.0%)	16 (33.3%)	0.57
	Enterococcus	13 (7.3%)	13 (10.1%)	0 (0%)	0.02
	CONS	20 (11.3%)	13 (10.1%)	7 (14.6%)	0.40
	HACEK	5 (2.8%)	2 (1.6%)	3 (6.3%)	0.12
	Other	16 (9.0%)	11 (8.5%)	5 (10.4%)	0.77
	Negative culture	17 (9.6%)	11 (8.5%)	6 (12.5%)	0.40
	Healed	10 (5.6%)	10 (7.8%)	0 (0.0%)	0.06
Indication	Uncontrolled infection	37 (20.9%)	19 (14.7%)	18 (37.5%)	<0.001
	Emboli	22 (12.4%)	17 (13.2%)	5 (10.4%)	0.62
	CHF/valve dysfunction	118 (66.7%)	93 (72.1%)	25 (52.1%)	0.01
Vegetation size (mm)		14.12 ± 5.74 (*n* = 110)	13.99 ± 5.8 (*n* = 78)	14.44 ± 5.66 (*n* = 32)	0.71
Operative data					
Valve type	Tissue	122 (68.9%)	85 (65.9%)	37 (77.1%)	0.15
	Mechanical	30 (16.9%)	20 (15.5%)	10 (20.8%)	0.40
	Repair	32 (18.1%)	26 (20.2%)	6 (12.5%)	0.24
Concomitant procedures		58 (32.8%)	46 (35.7%)	12 (25.0%)	0.18
	CABG	22 (12.4%)	20 (15.5%)	2 (4.2%)	0.34
	MV repair	2 (1.1%)	1 (0.8%)	1 (2.1%)	
	TV annuloplasty	16 (9.0%)	13 (10.1%)	3 (6.3%)	
	Ablation/excision of LAA	9 (5.1%)	6 (4.7%)	3 (6.3%)	
	Other	9 (5.1%)	6 (4.7%)	3 (6.3%)	

^†^ Abbreviations: BAV—bicuspid aortic valve; CABG—coronary artery bypass grafting; CHF—Congestive heart failure; CONS—coagulase-negative staphylococcus aureus; COPD—chronic obstructive pulmonary disease; DM—diabetes mellitus; EF—ejection Fraction; HACEK group—Hemophilus, Actinobacillus, Cardiobacterium, Eikenella, Kingella; HTN—hypertension; IVDU—intravenous drug user; LAA—left atrial appendage; MV—mitral valve; PVD—peripheral vascular disease; RHD—rheumatic heart disease; TV—tricuspid valve.

**Table 2:** Early and long-term results (univariate analysis).

		All (*n* = 177)	Simple (*n* = 129)	Complex (*n* = 48)	*p*-Value
In hospital	Operative mortality	21 (11.9%)	12 (9.3%)	9 (18.8%)	0.08
	Length of stay—days (median, interquartile range)	15 (10–22)	15 (9.5–22)	15 (11–21.75)	0.67
	ICU stay (median, days)	2 (1–4)	2 (1–3.25)	2 (1–6.75)	0.04
	Stroke	10 (5.6%)	8 (6.2%)	2 (4.2%)	0.73
	Pacemaker implantation	11 (6.2%)	5 (3.9%)	6 (12.5%)	0.07
	Acute renal failure	27 (15.3%)	16 (12.4%)	11 (22.9%)	0.08
	Prolonged ventilation	28 (15.8%)	15 (11.6%)	13 (27.1%)	0.01
Late	Wound infection	3 (1.9%)	3 (2.6%)	0 (0.0%)	0.56
	Re-endocarditis	4 (2.5%)	2 (1.7%)	2 (5.1%)	0.17
	Reoperation d/t re-endocarditis	1 (0.6%)	0 (0.0%)	1 (2.6%)	0.27
	Re-intervention for structural valve degeneration ^†^	6 (5.9%)	6 (8.2%)	0 (0.0%)	0.18

^†^ Rates are based on bioprostheses only (*n* = 101; simple = 73, complex = 29).

**Table 3:** Cox regression multivariate analysis for long-term mortality.

	*p*-Value	Hazard Ratio	95% C.I.	
			Lower	Upper
Complex	0.167	1.675	0.806	3.482
Age > 65	0.015	2.380	1.179	4.801
Female gender	0.042	1.933	1.023	3.656
Chronic renal failure/dialysis	<0.01	3.566	1.798	7.075
Mechanical valve	0.225	0.455	0.128	1.621
Diabetes mellitus	0.304	1.481	0.700	3.134

## Data Availability

The data presented in this study are available on request from the authors.

## Supplementary Materials

The following supporting information can be downloaded at: https://www.mdpi.com/article/10.3390/jcm14165870/s1.

## Abbreviations

The following abbreviations are used in this manuscript:

BAV	Bicuspid aortic valve
CABG	Coronary arteries bypass surgery
CHF	Congestive heart failure
CONS	Coagulase-negative staphylococcus aureus
COPD	Chronic obstructive pulmonary disease
DM	Diabetes mellitus
EF	Ejection fraction
HACEK group	Hemophilus, Actinobacillus, Cardiobacterium, Eikenella, Kingella
HTN	Hypertension
ICU	Intensive care unit
IE	Infective endocarditis
IVDU	Intravenous drug user
LAA	Left atrial appendage
MV	Mitral valve
PVD	Peripheral vascular disease
RHD	Rheumatic heart disease
SVD	Structural valve degeneration
VIV	Valve in valve
TF	Trans-femoral
TV	Tricuspid valve

## Appendix A

**Table A1:** Details of repeat cardiac procedures.

No	Surgery Date	Age	Simple/ Complex	Surgery	Re-do Procedure	Etiology	Date	Time Elapsed
1	6 April 2005	46	Simple	AVR + MVR bioprostheses	Re-do MVR bioprosthesis + TV annuloplasty	SVD	21 February 2012	7 years
2	25 April 2006	43	Simple	MV repair: resection of P2, part of P1, sliding plasty, annuloplasty	MVR mechanical	Early failure susp. Re-endocarditis	4 May 2006	9 days
3	31 May 2006	55	Simple	AVR Mechanical	Re-AVR mechanical	Paravalvular leak	11 June 2006	11 days
4	28 May 2006	48	Simple	MVR Mechanical + concomitant TV repair	re-do AVR mechanical + implantation of epicardial electrode	Late aortic regurgitation	22 July 2021	15 years
5	29 November 2006	77	Simple	AVR + MVR bioprostheses	VIV mitral	SVD	1 January 2022	16 years
6	7 March 2007	44	Simple	AVR bioprosthesis	Re-do CABG	IHD	15 February 2015	8 years
7	26 March 2008	66	Simple	AVR bioprosthesis + CABG	TF VIV Evolut R 23	SVD	19 February 2020	12 years
8	9 July 2009	56	Simple	MVR bioprosthesis + CABG+ closure of LAA	TF mitral VIV Sapien 3 26 + PCI to RCA	SVD	2 April 2020	11 years
9	23 May 2012	55	Complex	Aortic root replacement freestyle, MV repair: patch reconstruction of aorto-mitral continuity and anterior leaflet	Re-root replacement with biological composite graft	Re-endocarditis	16 January 2024	12 years
10	22 August 2012	57	Simple	MVR bioprosthesis + CABG	TF mitral VIV sapien 3 + PCI to SVG	SVD	19 February 2018	6 years
11	1 September 2014	53	Complex	AVR mechanical	Removal of pacemaker leads	Device-related right-sided endocarditis, normal prosthetic valve	10 November 2020	6 years
12	7 September 2015	66	Simple	MVR bioprosthesis + TV annuloplasty + lt.-sided cryo-ablation + Excision of LAA	Mitral VIV Sapien 3	SVD	18 April 2024	9 years
13	18 November 2020	60	Simple	MV repair: resection of posterior leaflet p2–3, annuloplasty + CABG	MVR bioprosthesis + TV annuloplasty	Early failure	10 December 2020	3 weeks
14	13 June 2022	47	Simple	MVR mechanical	CABG	IHD	4 July 2023	1 year

Abbreviations: AVR—aortic valve replacement; CABG—coronary artery bypass grafting; LAA—left atrial appendage; MVR—mitral valve replacement; SVD—structural valve degeneration; VIV—valve in valve; TF—trans-femoral; TV—tricuspid valve.
