# Supplementary material for: Surgery for Complex vs. Simple Native Left-Sided Endocarditis: Insights from an Extended Follow-Up on Survival, Recurrent Infection, and Valve Durability
Source: J Clin Med. 2025 Aug 20;14(16):5870. doi: 10.3390/jcm14165870 (PMC12387112; doi:10.3390/jcm14165870)

Stratification of long-term survival by surgical indication:

Overall Comparisons

	Chi-Square	df	Sig.
Log Rank (Mantel-Cox)	1.070	2	.586

Test of equality of survival distributions for the different levels of indication.

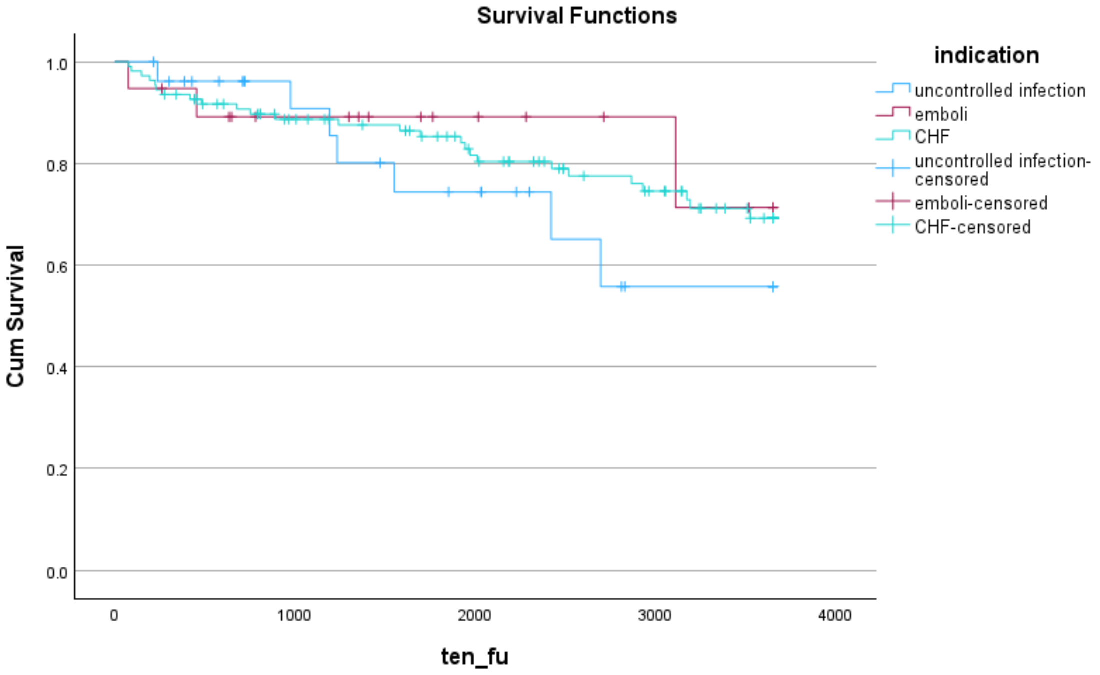

Supplement: Supplementary file 1 [file jcm-14-05870-s001.zip › jcm-3745584-supplementary.pdf]
